# Multi-disciplinary interventions for chronic pain involving education: A systematic review

**DOI:** 10.1371/journal.pone.0223306

**Published:** 2019-10-02

**Authors:** Shirdhya Joypaul, Fiona Kelly, Sara S. McMillan, Michelle A. King

**Affiliations:** School of Pharmacy and Pharmacology, Menzies Health Institute Queensland, Griffith University, Queensland, Australia; York University, UNITED KINGDOM

## Abstract

**Background:**

There have been growing recommendations to include education in multi-disciplinary interventions targeting chronic pain management. However, effects of this strategy on short- and long-term self-management of chronic pain, remain largely unexplored.

**Objectives:**

1. To provide an updated overview of studies that report on the impact of patient education in multi-disciplinary interventions, on self-management of chronic pain; 2. To explore associations between education and chronic pain self-management techniques; and 3. To identify the format and duration of suitable chronic pain interventions targeted at patient self-management.

**Methods:**

*Design*: Narrative systematic literature review of randomised or controlled study designs. *Data Sources*: PubMed, CINAHL, EMBASE, PsycINFO. *Participants*: Adult patients with chronic pain of any aetiology participating in multi-disciplinary programs that included education. *Main outcome measures*: Assessments of level of pain, function, quality of life, self-efficacy, self-management, and any other relevant assessments. *Study Appraisal and Synthesis Methods*: PRISMA guidelines, Cochrane Risk of Bias tool, and TIDieR model.

**Results:**

Database searching identified 485 potential papers. After removal of duplicates, and irrelevant articles by title and abstract, 120 full-text articles were reviewed and 27 studies were included in this systematic review. Studies were predominantly from the United States (n = 8; 29.6%). Over one hundred outcome measures were identified across all studies, with significant variation also observed in terms of how chronic pain duration was defined, and how education was delivered to participants. Overall, positive benefits of education were reported.

**Conclusions:**

Education, as part of multi-disciplinary programs, is likely to improve self-management and self-efficacy in people with chronic pain of any aetiology. Heterogeneity in terms of: chronic pain duration; educational resources; healthcare professionals; and outcome measures, were identified as limitations. Further research, in the form of Randomised Controlled Trials addressing these limitations, is recommended.

## Background

Chronic pain is pain that lasts or recurs for more than three months [1–3]. Globally, prevalence of chronic pain has been reported as 37.3% of the population in developed countries and 41.1% in developing countries [4]. In Australia, chronic pain is the fourth most common chronic condition [1], affecting almost 20% of the population [5]. Chronic pain is complex in terms of aetiology and management approaches [6–8], and despite the existence of pharmacological therapy [9, 10], remains highly resistant to treatment [5, 10]. Chronic pain significantly and negatively impacts upon individual lives, leading to physical disability, mental health problems and long waitlists for specialist health services, as well as economic costs to health services, patients and the community [1, 4, 11–14]. A sustainable solution is needed to reduce these negative impacts and promote effective self-management, with increased recognition of the role of public education around pain and its management, and co-ordinated multi-disciplinary (MD) care [1, 9].

Public education promotes patient awareness of the somatic, psychological and social aspects of chronic pain [1, 6–9], and supports self-management with the aim to improve quality of life [1]. Education alone can have a limited impact on chronic pain [15–24], yet when combined with MD programs, there seems to be additional benefits for chronic pain management, such as increased patient confidence and self-efficacy [25–27]. MD care refers to the collaboration of healthcare professionals from a range of disciplines to deliver comprehensive patient care that meets the needs of the individual [28]. MD programs designed for chronic pain management generally include patient education and other services or care, such as medication reviews, self-management programs, allied health and community-based services, in various delivery modes, such as face-to-face, teleconferencing, and/or internet-based sessions [1, 9]. Including education in MD programs has been associated with medication optimization, reduced pain catastrophizing, and reduced utilisation of secondary care services [9, 25, 26, 29]. However, research has highlighted the need for Randomised Controlled Trials (RCTs) to confirm the immediate and sustained impact of these programs on health outcomes [25, 26].

There has been limited reporting of empirical evidence of MD interventions; questions remain regarding the efficacy of individual versus group education and the optimal combination of educational topics [1, 27, 30–32]. Although there are some randomised and/or controlled studies [33–59] and reviews in this area [27, 30–32], they have specific limitations. The most detailed systematic review to date was published by Scascighini *et al*. in 2008 [27]. MD programs, with or without education components, were reported to be more effective than no treatment or standard medical treatment, in participants with chronic, non-specific musculoskeletal pain [27]. The authors concluded that incorporation of once-weekly patient education sessions in MD programs was a superior strategy compared to other medical treatment [27]. A strength of the review was the exclusion of non-RCT studies, however, limitations included: an incomplete reporting of the search strategy; a narrow scope focussing solely on cognitive-behavioural and psychological graded interventions; and limited assessment of methodological quality and risk of bias assessments of included studies [27]. More recent meta-analyses were restricted to small numbers of included papers and the investigation of education as a solitary intervention rather than as part of MD programs [31, 32]. Another narrative review around preventive interventions focused on post extremity trauma, that is, one pain type only [30].

Questions remain on how to best incorporate education into MD programs for chronic pain. Such knowledge remains critical in an era when the prevalence of chronic pain is predicted to rise with an ageing population [5, 29]. The aim of this systematic literature review was to update and extend Scascighini *et al*.’s review [27]. This included: 1. Using a well-defined and comprehensive search strategy to identify studies that report the impact of patient education in a broad range of MD interventions; 2. Exploring for associations between education and chronic pain self-management techniques; and 3. Identifying format and duration of suitable chronic pain interventions targeted at self-management from methodological quality and risk of bias assessments. The findings of this review will inform researchers, healthcare professionals and/or people experiencing chronic pain about the current evidence of MD interventions, as well as highlight critical aspects to include in future programs alongside realistic expectations of effectiveness.

## Methods

A systematic literature review was performed according to the methods outlined in the Preferred Reporting Items for Systematic Reviews and Meta-Analyses (PRISMA) guidelines [60]. PRISMA guidelines constitute an evidence-based protocol for developing and reporting narrative systematic reviews and meta-analyses [60], and are recommended by the Cochrane community and the EQUATOR network when reporting database searches in systematic reviews [61, 62]. Prior to commencing the systematic review, all authors agreed to relevant definitions and a robust search strategy and study selection process (see Supporting information); two attempts at contacting Scascighini *et al*. [27] for a detailed search strategy were unsuccessful. No protocol exists for this work.

### Definitions

For the purpose of this review, the definition of chronic pain was: pain of any aetiology persisting for more than three months [3]. Interventions were deemed MD if they involved collaboration of healthcare professionals from a range of disciplines to deliver comprehensive care to the patient [28]. Education was defined as instructions to inform participants about self-management and/or medication-taking techniques for their chronic pain. The education could be delivered via a range of modalities, including face-to-face, teleconferencing, internet-based sessions and/or use of other multimedia content. Participants included persons with chronic pain who consented to take part in the research, such as those who volunteered, persons who were referred to pain clinics or health centres, individuals who were on sick leave from work, or persons living in retirement communities. Study designs included in this review were RCTs and/or other randomised or controlled study designs (e.g. cluster randomised trials).

### Data sources

A specialist librarian was consulted on three occasions for advice around, and refinement of, the search strategy (see S2 Appendix). Health databases searched were CINAHL, EMBASE, PubMed and PsycINFO, using a combination of Medical Subject Heading (MeSH) terms (e.g. “chronic pain,” “health education,” “multidisciplinary care team,” and “interdisciplinary treatment approach”) and text words (e.g. “self care”). These databases represent the four most appropriate databases that were likely to produce a broad range of peer-reviewed literature across the disciplines of medicine, nursing, psychology and allied health. The search was conducted on five occasions from mid-2017 to August 2019, with the search strategy consistently adopted across all databases to ensure identification of eligible studies. The final list of studies was exported to a referencing management system (EndNote^®^) in August 2019.

### Eligibility criteria and study selection

The following inclusion and exclusion criteria were established (Table 1).

**Table 1:** Inclusion and exclusion criteria.

Inclusion Criteria:
1. Multi-disciplinary interventions of any duration.
2. Study participants included adults only (minimum of 18 years). If studies included children younger than 18 years, these were considered if they reported results specific for adults.
3. Language was limited to those understood by the authors–English, French, Dutch and German.
4. Chronic pain of any aetiology. Studies involving both acute and chronic pain were considered if they reported results specific to chronic pain.
5. The intervention was a randomised or controlled study design that involved an educational component of any form (e.g. lectures, online links, leaflets, apps and books) about any topic (e.g. medication management, pain control, understanding pain, etc.)
Exclusion Criteria:
1. Interventions were not multi-disciplinary.
2. Identified articles had no element of educational intervention for patients and/or were only pharmacological in nature.
3. Studies involving only acute pain.
4. Studies involving only cancer pain.
5. Study participants involving children and adolescents only (younger than 18 years).
6. Languages other than English, French, Dutch and German.

Eligibility for inclusion/exclusion was first assessed via independent duplicate manual screening of article titles and abstracts (S.J. and S.M.). Disagreements were resolved by discussion and consensus with a third author (M.K.). The same process was undertaken when reviewing all relevant full-text articles. A supplementary search method, involving a review of reference lists of included full-text articles (i.e. snowballing), was adopted.

### Data extraction

Information was extracted into a Microsoft Excel^®^ spreadsheet and included characteristics such as country, sample size, number of chronic pain types and/or sites, and members of the MD team. The outcome measures were noted as total number of outcome measures used, those that showed significant results and whether such measures were validated. Information extracted into a separate Microsoft Word^®^ document included more detailed description of the individual interventions including type and delivery mode, as well as study setting, patient details and age range, findings and follow-up. The objectives, randomisation and blinding procedures, as well as group allocation details, were also noted.

### Risk of bias and quality of reporting

Risk of bias and quality of reporting assessments were undertaken by the first author (S.J.), following concurrent independent testing (S.J. and M.K.) of the first six included articles against a checklist developed from the Cochrane Collaboration Risk of Bias tool [63–65] and the Template for Intervention Description and Replication (TIDieR) [66, 67]. Where articles lacked information, the respective study protocols were consulted. These steps ensured the completeness of reporting and helped determine the reliability and replicability of included studies [63–67]. Any disagreements were resolved through discussion between all authors.

### Data analysis

Given the heterogeneity of study methods and outcome measures across all included studies as well as the broad nature of the review, statistical comparisons between studies were not possible. Hence, a narrative description of the data was adopted as a suitable method to meet the review aims [68]. Other narrative studies published in evidence-based journals and databases such as PLoSONE [69] and Cochrane [61], are known to have used the same methodology.

The authors systematically analysed all reported outcomes across all included studies. Thematic analysis was performed from all outcomes and only those findings that aligned with the objectives of the work, have been reported in this paper.

## Results

### Search results

**Fig 1:**
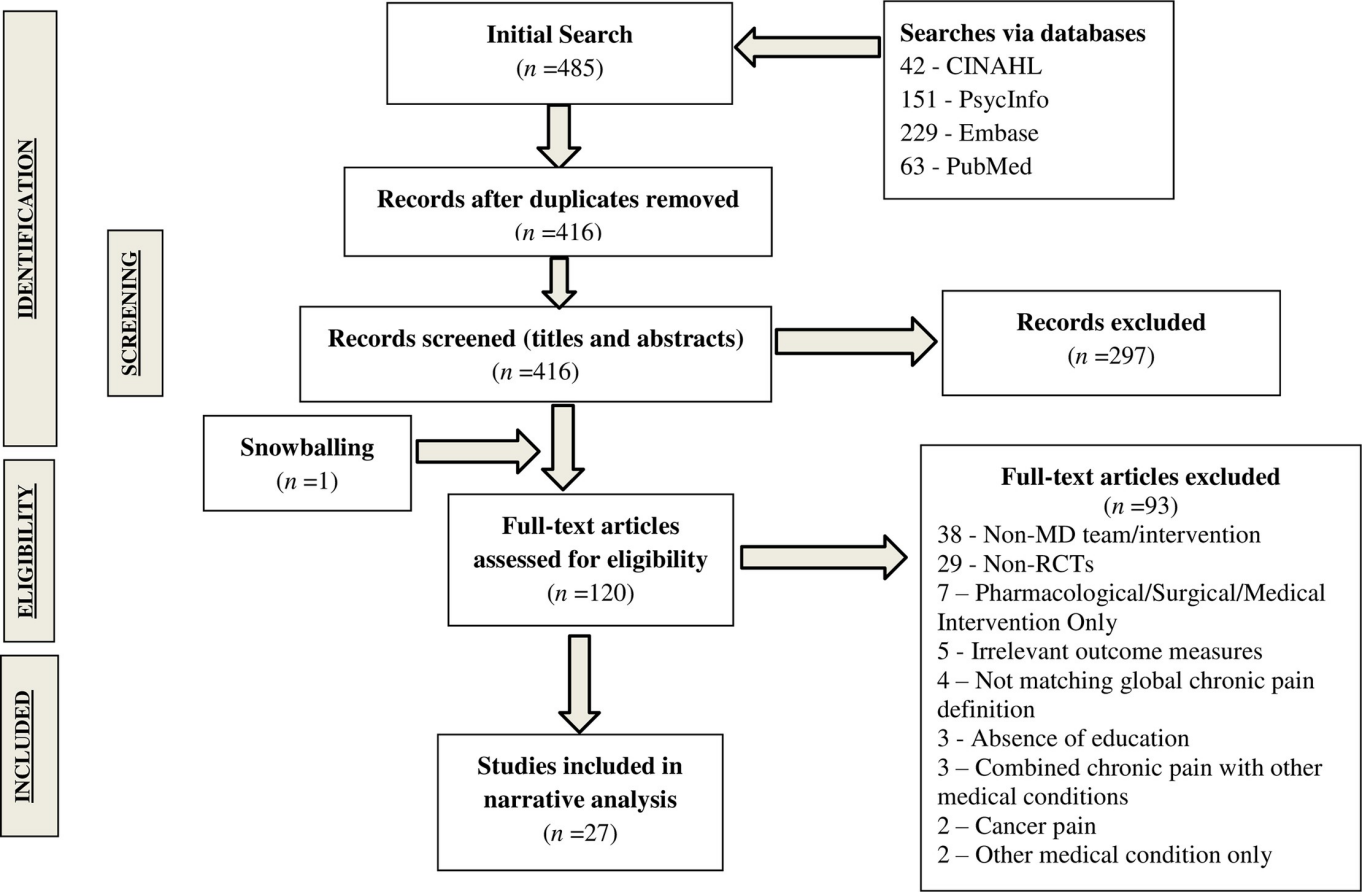
PRISMA flowchart of study inclusion.

The search strategy identified 485 records of which 69 duplicates were removed. An additional 297 records were excluded through title and abstract screening, with a total of 120 full-text articles retrieved for further evaluation. Twenty seven studies met the inclusion criteria (see Fig 1) [33–59]. A concise overview of data from these included studies is provided in Tables 2 and 3.

**Table 2:** Study characteristics.

Characteristic		No. of Studies	Characteristic		No. of Studies
***Country***	United States of America	8	***Sample Size***	<30	1
	Australia	4		30–100	13
	Germany	3		100–300	11
	Denmark	2		>300	2
	Norway	2			
	Spain	2			
	Other^#^	6			
Chronic Pain Site*	Multiple sites	17	***Chronic Pain Minimum Duration***	3 months	14
	Back	7		6 months	7
	Neck	2		Specified as “chronic pain”	6
	Head	1			
***Types of Health Professionals***^*§*^	Physiotherapist	17	***Number of Types of Health Professionals***	2	3
	Psychologist	17		3	8
	Nurse	9		4	1
	Physician/General Practitioner	6		5	2
	Pain Specialist	6		7	2
	Research Health Assistant	4		Other^^^	11
	Technique Instructor	2			
	Pharmacists	2			
	Other^o^	5			
	Not specified	10			
***Design***	One intervention and one control group	22	***Delivery Mode of Intervention***	Individualised only	11
	Two interventions and one control group	4		Group only	9
	One intervention and two control groups	1		Individualised and Group	7
				Face-to-face only	18
				Face-to-face and telephone	5
				Telephone only	1
				Unsure^~^	3
***Elements of Interventions***	Cognitive Behavioural Strategies^¤^	21	***Number of Outcome Measures***^***Δ***^ ***Utilised in Included Studies***	≤ 3	4
	Physical Activity^¤^	11		4–6	12
	Medication Monitoring/Optimisation	7		7–9	6
	Back School^γ^	2		≥ 10	5
	Manual Therapy	1			
	Acupuncture and massage	2			
	Supply of take-home materials^¤^ and/or homework	23			
***Follow-Up Period***	< 3 months	5			
	3–12 months	18			
	18 months	1			
	None	3			

^#^ One study from each of the following countries: Brazil, Iran, Netherlands, Portugal, Scotland and United Kingdom

*Various terminology as used to specify pain type across all studies (e.g. “chronic widespread” and “chronic non-malignant” to describe widespread pain)

^ο^One study with each of the following professionals: Acupuncturist, Massage Therapist, Nutritionist, Social Worker and Surgeon

^§^Numbers do not add up to 27 as studies had more than one type of health professional

^^^Of the 27 studies included, 11 did not specify which healthcare professionals were involved. One mentioned an “activities director”, one mentioned “statistician”, one mentioned “therapist” with no definition of the term, one mentioned “Occupational Therapy and Other Medical Staff”, one mentioned “Primary Care Provider and Behavioural Health Specialist” with no clarification of the term and six were incomplete in their mention of all types of healthcare professionals constituting the team

^~^ Delivery mode, as mode of communication, was not specified

^¤^ Examples of Cognitive Behavioural strategies are: mindfulness and relaxation; examples of physical activity are: aerobic exercises, aquatic exercises and yoga; examples of take-home materials are: books and smartphones

^γ ^Back School consists of an educational program merged with skills acquisition (incl. physical activity)

^Δ^ A wide range of outcome measures (n~111) were used. Eleven outcome measures crossed over across studies. These included: Rolland-Morris Disability Questionnaire (RDQ), Visual Analog Scale (VAS) and Health Survey Short Form-36 (SF-36), each crossing over in four studies; Tampa Scale for Kinesiophobia (TSK), Hospital and Anxiety Depression Scale (HADS), Numerical Rating Scale for pain (NRS) and Pain Self-Efficacy Questionnaire (PSEQ), each crossing over in three studies; and Fibromyalgia Impact Questionnaire (FIQ), Brief Pain Inventory (BPI), Neck Disability Index (NDI) and Fear Avoidance Beliefs Questionnaire (FABQ) each crossing over in two studies.

**Table 3:** Summary of included studies (PICO).

Author/s (Reference)	Sample (Pain Type, Number)	Intervention/s and Control	Education Topics	Primary Outcome(s)
**Abbasi et al. (2012) [33]**	Chronic low back pain >6/12 months (n = 33).	Group 1: Spouse-Assisted (n = 10); Group 2: Patient-Oriented (n = 12). Seven weekly 2h sessions, private psychiatrist and physiotherapist sessions (Groups 1 and 2). Group 3: Control; ordinary medical care (n = 11).	• Physiology • Psychology	• Roland-Morris Disability Questionnaire (RDQ) • Visual Analogue Scale (VAS)
**Anderson et al. (2007) [34]**	Chronic widespread pain > 3/12 months and > 10 Tender Points (n = 52).	Treatment group (n = 19)—Eleven 4-h sessions over one month. Training with physiotherapist over 1.5 years; Control group (n = 26): Treatment as usual.	• Exercise • Cognitive Behavioural Therapy (CBT) • Relaxation • Body awareness	• Work status • Global Physiotherapeutic Examination (GPE-52) • Tender Points (TePs) • VAS
**Bair et al., 2014 [35]**	Chronic and disabling musculoskeletal pain pain score ≥ 4 and Roland Morris Disability score ≥ 7 (n = 241).	Stepped Care Intervention (n = 121) - Twelve phone calls. Psychologist, physicians and nurses; Usual Care (n = 120)–Treatment as usual.	• Analgesic treatment • Self-management strategies • Mental health • CBT • Musculoskeletal pain	• RDQ • Brief Pain Inventory (BPI) • Graded Chronic Pain Scale.
**Basler et al., 1996 [36]**	Migraine and/or tension headache (n = 88).	Intervention group (n = 50)—Twelve group sessions with psychologists; Control group (n = 38)—Treatment as usual.	• Pain experience • Pain cycle • Self-control • Pain medications	• Pain diary
**Becker et al., 2000 [37]**	Chronic non-malignant pain (n = 167).	Group 1 (n = 56)—MD treatment with pain specialists; Group 2 (n = 58)—Treatment by general practitioner and pain specialists; Control group 3 (n = 53)–Treatment as usual.	• Physiology and psychology of pain • Pain management strategies • Analgesic treatment • Biomechanics	• VAS • Health Survey Short Form-36 (SF-36) • Hospital Anxiety and Depression Scale (HADS) • Psychological General Well-being Scale (PGWB)
**Beltran-Alacreu et al., 2015 [38]**	Neck pain ≥ 12/52 weeks (n = 45).	Group 1 (n = 15)–Manual therapy and education sessions; Group 2 (n = 15)—Shorter manual therapy and exercises; Control Group (n = 15)—Manual therapy sessions only. Involvement of physiotherapists and physician across all groups.	• Self-management strategies • Biobehavioural strategies.	• Neck Disability Index (NDI)
**Burke et al., 2016 [39]**	Chronic pain (n = 712).	Experimental (n = 485) - 3h session with pain consultant, psychologist and physiotherapist (n = 485); Treatment as Usual (n = 227)—Normal wait list procedure	• Self-review strategies • Self-management strategies • Life engagement strategies	• Patient Screening Questionnaire (PSQ) • Chronic Pain Acceptance Questionnaire (CPAQ) • World Health Organisation QOL-Brief Scale (WHOQOL-BREF) • Pain-related health knowledge and beliefs • Symptom exaggeration
**Chao et al., 2019 [59]**	Chronic pain >3/12 months (n = 61).	Group 1 (n = 41)–Twelve weekly 2-h sessions with health educator, program coordinator, clinical pharmacist, certified yoga and movement instructor, mindfulness instructor, certified massage therapist and nutritionist. Additional one-on-one MD pain management. Group 2 (n = 20)–Treatment as usual until enrolment after Group 1.	• Neuroscience • Stories, successful treatments, and self-care • Physical movement • Medication (incl. naloxone training) • Meditation/mindfulness • Self-massage • Nutrition	• Pain interference • Average pain intensity • Anxiety • Depressive symptoms • Physical functioning • Social satisfaction • Global mental health • Global physical health • Pain catastrophizing • Pain Self-Efficacy Questionnaire (PSEQ)
**Cherkin et al., 2001 [40]**	Low back pain (n = 262).	Acupuncture Group (n = 94)–Ten Traditional Chinese Medical practices over 10 weeks with licensed acupuncturists. Massage Group (n = 78)–Ten massage sessions over 10 weeks with licensed massage therapists. Self-Care Group (n = 90)—Supply of educational materials (one book and two videotapes).	• Exercise • Self-management strategies	• Symptoms scales • Dysfunction scales
**Corson et al., 2011 [41]**	Musculoskeletal back pain and/or arthritic pain and/or neck pain and/or joint pain ≥ 12/52 weeks, Chronic pain grade (CPG) score ≥ 4/10 and RDQ ≥ 6/24 (n = 365).	Intervention Group (n = 169)—MD program to optimise patient outcomes and self-management. Control (n = 196): Treatment as usual.	• Pain, function and mental disorders • Chronic Care model • Decision-making • Setting functional goals • Treatment	• Pain Process Checklist (PCC)
**Cramer et al., 2013 [42]**	Non-specific Neck Pain ≥ 12/52 weeks and ≥ 5/7 days, VAS ≥ 40 mm (n = 51).	Iyengar Yoga Group (n = 25)–Nine 90-min sessions with instructor, physiotherapist and psychologist. Exercise Group (n = 26)—Self-care manual with seated exercise instructions.	• Yoga • Seated exercise	• Neck pain intensity (100mm VAS)
**Ersek et al., 2008 [43]**	Any non-cancer pain > 3/12 months, average pain >2 in past week on 0–10 scale (n = 256).	Self-Management Group (n = 133)—Seven weekly 90-min sessions. Control Group (n = 123)—Two books: *The Chronic Pain Workbook* and *Managing Your Pain Before It Manages You*.	• Basic principles of chronic pain • Exercise • Engaging in pleasant, meaningful activities • Pacing • Challenging negative thoughts • Dealing with flare-ups and setbacks • Nondrug and drug therapies	• RDQ
**Gallagher et al., 2013 [44]**	Disruptive pain ≥ 3/12 months (n = 79).	Intervention Group (n = 40) - 80-page booklet with 11 short stories on pain biology to read over three weeks. Control Group (n = 39) - 80-page booklet with 11 sections on CBT to read over three weeks.	• Pain biology • CBT	• Pain Biology Questionnaire (PBQ) • Pain Catastrophising Scale (PCS)
**Heutink et al., 2012 [45]**	Spinal Cord Injury (SCI) with neuropathic pain ≥ 6/12 months; pain intensity ≥ 40 on Chronic Pain Grade scale in previous week (n = 61).	Intervention Group (n = 31)—Ten 3h sessions over 10 weeks with psychologist, physiotherapist and nurse. Waiting List Group (n = 30)—Treatment as usual.	• BioPsychoSocial (BPS) model • SCI and Chronic Neuropathic Spinal Cord Injury pain (CNSCIP) • Rehabilitation, movement and pain • Assertiveness and communication about pain • Pain, mood and stress • Social aspects of pain	• Chronic Pain Grade Questionnaire (CPGQ)
**Jay et al., 2016 [46]**	Chronic musculoskeletal pain in ≥ 1 region of upper back, lower back, neck, shoulders, elbows, and hands/wrists; pain intensity ≥ 3 on VAS, pain frequency ≥ 3 days in last week, pain ≥ 3/12 months (n = 112).	Physical-Cognitive Mindfulness Training (n = 56)– 10-week program with specialist trainers and psychologist Reference Group (n = 56) - 10-week ongoing company initiatives to reduce musculoskeletal pain at work.	• Pain • Fear-avoidance • Catastrophizing • Exercise • CBT • Mindfulness	• Fear Avoidance Beliefs Questionnaire (FABQ)
**Keller et al., 1997 [47]**	Chronic Low Back Pain (n = 64).	Treatment Group (n = 35)—Eighteen individualised 30min training sessions and 18 2h group meetings with physiotherapist, psychologist and pain specialist. Waiting List Control Group (n = 29)—Treatment as usual.	• Vicious cycle of pain • Avoidance • Demoralisation and dysphoric mood • Treatment methods towards gaining self-control • Pain-related behaviour	• Pain Frequency • Typical Pain Intensity
**Kerns et al., 2014 [48]**	Back pain ≥ 6/12 months, Score ≥ 4 on pain scale over past week (n = 128).	Tailored CBT Group (n = 68)–Ten 60min individualised pain coping modules with motivational enhancement. Standard CBT Group (n = 60)–Ten 60min individualised pain coping modules maximally mismatching those in TCBT; no motivational enhancement.	• Exercise • Relaxation • Cognitive control • Body mechanics • Pacing • Task persistence • Assertiveness • Asking for help	• Adherence/goal accomplishment • Treatment “dose” • Treatment engagement
**Kristjánsdóttir et al., 2013 [49]**	Chronic Pain >6/12 months (n = 140).	4-week MD rehabilitation pre-intervention for both groups. Smartphone Group (n = 48)—three diary entries/day on smartphone; SMS reminders of self-management strategies daily; and guided mindfulness exercises on phone. Control Group (n = 64)—No smartphone intervention.	• Pain mechanisms • Self-management strategies • CBT	• PCS
**Martin et al., 2012 [50]**	Widespread fibromyalgia pain ≥ 3 months, pain on palpation in ≥ 11 of 18 tender point sites (n = 180).	Experimental Group (n = 90)—Six-week biweekly MD sessions (PSYMEPHY) with physician, psychologist and physiotherapist; Standard pharmacological treatment. Control Group (n = 90)—Standard pharmacological treatment only.	• Fibromyalgia • Pacing • Breathing • Positive thinking • Assertiveness • CBT • Warming and stretching exercises • Communication skills with health professionals	• Fibromyalgia Impact Questionnaire (FIQ)
**Nicholas et al., 2013 [51]**	Non Cancer Pain ≥ 6/12 months and at one or more major sites, score ≥ 22 in Rowland Universal Dementia Assessment Scale (n = 141).	Pain Self-Management Group (n = 49)—CBT and education with nurse, physiotherapist and psychologist. Exercise Attention Control Group (n = 53)—Stretching and aerobic exercises with physiotherapist and psychologist only. Eight 2h bi-weekly sessions for 4 weeks for both PSM and EAC. Waiting List Group (n = 39)–Treatment as usual.	• CBT • Self-efficacy • Autonomy • Pain medications	• Modified RDQ
**Pires et al., 2015 [52]**	Chronic low back pain ≥ 3/12 months (n = 62).	Education Group (n = 30)–Two (90min) education sessions and 12 (30 to 50min) bi-weekly sessions of aquatic exercise over 6 weeks. Control Group (n = 32) -Aquatic exercise only.	• Pain neurophysiology • Psychosocial factors related to pain • CBT • Flare-up management • Pacing	• VAS • Quebec Back Pain Disability Scale (QBPDS)
**Ribeiro et al., 2008 [53]**	Chronic low back pain > 3/12 months (n = 60).	Back School Intervention Group (n = 29)–Five weekly sessions with rheumatologist and physical therapist. Control Group (n = 31)—Four medical visits with rheumatologist only.	• Anatomy and physiology of spine • Causes and treatment of chronic low back pain • Ergonomics • Exercise • Relaxation	• Schober’s Test • VAS • SF-36 • RDQ • Beck Depression Inventory • State-Anxiety Inventory (STAI)
**Ryan et al., 2010 [54]**	Chronic low back pain >3/12 months (n = 38).	Education Only (n = 18)– 2.5h session only. Education and Exercise (n = 20)—Education and six exercise classes over eight weeks.	• CBT • Pain biology • Beliefs and attitudes about back pain • Fear avoidance and harm beliefs • Self-efficacy	• NRS • RDQ
**Smith et al., 2016 [55]**	Chronic non-cancer pain >3/12 months (n = 211).	Group Assessment Group (n = 104)– 5h assessment provided by physiotherapist and either nurse or psychologist. Individual Assessment Group (n = 107)—Three 1h interviews with pain specialist, psychologist and physiotherapist.	• Neurophysiology of pain • Medication management • Red flags • Nutrition • Physical activities • Pain flare-up and its impact on emotion, cognition, behaviour, occupation and social functioning • Relaxation	• BPI • PSEQ • Kessler 10 (K10)
**Sullivan et al., 2017 [56]**	Non cancer pain ≥ 3 months in past 6, use of opioids on ≥ 45 days in previous 90 days (n = 35).	Opioid Taper Support Group (n = 18)—Adjust/initiate medications; motivational interviewing on opioid tapering; viewing short videos; relaxation and pacing. Usual Opioid Prescribing Care (n = 17): Treatment as usual.	• Self-management/self-efficacy strategies (with CBT) • Dose-related health risks • Practical and psychological barriers	• Mean daily morphine- equivalent opioid dose
**Turner-Stokes et al., 2003 [57]**	Chronic pain >6/12 months (n = 126).	Group Treatment (n = 73)—MD CBT and physiotherapy program run over one afternoon for eight weeks by psychologist and physiotherapist. Individual Treatment (n = 53): Same as group but delivered by psychologist and physiotherapist for one hour every other week, over 8 weeks.	• CBT • Relaxation • Exercise • Pacing	• West Haven–Yale Multidimensional Pain Inventory (WHYMPI) • Beck Depression Inventory (BDI)
**Uebelacker et al., 2016 [58]**	Chronic pain ≥ 6/12 months, Brief Pain Inventory scale ≥ 5, NRS pain severity ≥ 4, elevated depressive Quick Inventory of Depression Symptoms (QIDS) ≥ 9 (n = 23; HIV+).	HIV-Pain And Sadness Study (n = 11)—Discussion of medical strategies to tackle individual’s symptoms. Health Education Control (n = 12)—Five telephone sessions based on individual’s choice of education topic. Both groups were run by Behavioural Health Specialists and Primary Care Providers (unspecified) every 2 weeks for 30-50minutes.	• Nature of chronic pain • Depression • Nutrition • Cold and flu • Cancer • Diabetes • Heart health • Complementary medicines • Caffeine • Exercises	• Pain-related interference with functioning (Brief Pain Inventory–interference scale BPI-I)

### Characteristics of identified randomised and/or controlled studies

Findings from the 27 included studies that aligned with the objectives of this work are summarised below.

#### 1. Participants

The number of study participants ranged from 23 [58] to 365 [41] across the 27 included studies. The minimum reported age of participants was 18 years [42] and the maximum reported age was 88 years [43]. One study did not specify participant age range of adult participants [55]. Most studies (n = 18) had more female than male participants [33, 34, 36–40, 42–44, 47, 50–54, 56, 57]; two studies only involved female participants [46, 49] and one did not specify gender distribution [55].

Intervention participants reported a diverse duration of chronic pain and its location or site/s. Fourteen studies reported a minimum chronic pain duration of three months [34, 38, 41–44, 46, 50, 52–56, 59], while seven studies included participants reporting pain for at least six months [33, 45, 48, 49, 51, 57, 58]. Six studies specified chronic pain, without an explicit pain duration for all participants [35–37, 39, 40, 47]. Participants with one pain site were addressed in ten studies [33, 36, 38, 40, 42, 47, 48, 52–54]. Of these, seven studies visited back pain [33, 40, 47, 48, 52–54], two studies visited neck pain [38, 42], and one study visited headaches [36]. The remaining 17 studies included participants with a mix of pain aetiologies and sites [34, 35, 37, 39, 41, 43–46, 49–51, 55–59].

#### 2. Interventions

Included studies were predominantly conducted in the United States (n = 8) [35, 40, 41, 43, 48, 56, 58, 59], Australia (n = 4) [39, 44, 51, 55], and Germany (n = 3) [36, 42, 47], with two studies each from Denmark [37, 46], Spain [38, 50], Norway [34, 49], and the United Kingdom [54, 57]. Education was didactic across all studies and was facilitated by a collaboration of healthcare professionals from at least two disciplines. Physiotherapists, psychologists and nurses constituted most MD teams across all included studies.

All 27 studies involved more than one educational topic either as part of the active intervention or usual care or both. Education around physical activity was most prominent (n = 23) [33–35, 37–40, 42, 43, 45–55, 57–59], followed by cognitive behavioural strategies (n = 22) [33–39, 43–52, 54–58]. While not explicitly stated, two other studies were likely to have involved exercise education, for example on “training to maintain gains” [56] or ergonomics [46], and three additional studies were likely to have used cognitive behavioural aspects [40, 41, 59]. Education about medications and optimisation of analgesic treatment were included in eight studies [35, 37, 49, 51, 55, 56, 58, 59], with advice on alternative pain management strategies also part of the medication management training in these studies. Education about nutrition was included in one study [59].

The didactic mode of education delivery across all studies was in the form of lectures or seminars, with 25 studies also reporting the provision of at least one supplementary educational tool to participants [33–49, 51, 52, 54–59]. These additional tools included personalised health plans (n = 9) [34, 37, 43, 48, 49, 55–58], regular (e.g. daily/biweekly) telephone calls to participants (n = 9) [35, 37, 41, 43, 44, 55, 56, 58, 59], written materials (n = 9) [33, 35, 38, 39, 42, 55, 57–59] and books (n = 7) [40, 43–45, 51, 54, 56]. Four studies provided recorded materials [40, 43, 47, 56], with two studies each providing tapes [40, 47] and CDs [43, 56], and one study providing videos [56]. Three studies used emails as an additional educational tool [41, 44, 46], three studies used diaries [36, 42, 49] in the form of journals (n = 2) [36, 42] and smartphone diaries (n = 1) [49]. Pictures and metaphors [52], diagrams and drawings [54], text messages [49], computerised registers [41], and webpages [49] were each used in a single study. Twenty-three RCTs provided homework activity or take-home materials to participants [33–36, 38–45, 47–51, 53–58].

Duration of education delivery varied from a minimum of three hours on a single day (n = 1) [39] to a maximum of once-weekly sessions over a period of 18 months (n = 1) [34]. Eleven studies provided education on an individualised basis [35, 37, 38, 40, 41, 44, 46, 48, 49, 56, 58]; eight studies involved group sessions [36, 39, 45, 50–54]; and eight combined individual and group education [33, 34, 42, 43, 47, 55, 57, 59].

The team involved in the delivery of the education content across all studies was mostly comprised of physiotherapists (n = 17) [33, 34, 37–42, 45, 47, 48, 50–53, 55, 57] and psychologists (n = 17) [33, 35–37, 39, 41–43, 45–48, 50, 51, 55, 57, 58]. Nurses (n = 9) were the third most common health professionals across all studies [35, 37, 41, 43, 45, 48, 49, 51, 55]. Health professionals in studies involving medication training were: nurses [35, 37, 49, 51], anaesthesiologists [33, 36, 37], general practitioners (GPs) [37], pain medicine specialists [55, 56], pharmacists [35, 59], physician assistants [56], and internal medicine physicians [58]. Only five studies described four or more different types of healthcare professionals as part of their MD team [33, 35, 37, 41, 55]; 11 studies were unclear on the actual roles/types of healthcare professionals involved [34, 43, 44, 46, 49, 52, 54, 56–59].

Participant check-ins or monitoring periods between active intervention sessions varied widely, ranging from at least daily checks [49] to outcome checks every two months [41]. These checks all preceded actual follow-up periods. Three of the 27 studies did not involve follow-up periods [41, 42, 46]. Of the remaining 24 studies, the maximum duration of follow-up was 18 months [34], with three to 12 months follow-up most commonly reported. Follow-up results were generally positive or showed no change compared to results at intervention completion. Fourteen studies adopted an intention-to-treat analysis in their methodology [33–35, 38, 40, 42, 43, 45, 46, 48, 49, 51, 52, 56].

### 3. Outcomes

Intervention outcomes and effectiveness were generally evaluated immediately after intervention conclusion, followed by re-evaluation at follow-up. The majority of studies (n = 23) showed statistically significant results (p<0.05 and/or large effect size) for one or more outcome measures [33–40, 42–47, 49–54, 56, 58, 59].

A total of 111 outcome measures were identified across all studies. Forty-two measures appeared once only and could not be compared between studies due to lack of similarity and uncertainty around validity. More than half of the included studies (n = 15) [34–37, 39, 40, 43, 47, 51, 53–58] used combinations of standardised, validated outcome measures (e.g. Tampa Scale for Kinesiophobia; TSK) with non-standardised, non-validated measures (e.g. distance walked in six minutes along corridor). Six studies had outcome measures tailored specifically to the research [36, 40, 47, 51, 53, 56]. None of the included studies used solely non-validated measures.

The number of outcome measures used per study ranged from one [46], to a maximum of 11 [47, 56], with no two studies using the same combination. Improvement in pain and mobility were measured in all 27 studies. These were mostly reported as scores from the Rolland Morris Disability Questionnaire (RDQ; n = 7) [33, 35, 41, 43, 51, 53, 54], the Visual Analog Scale (VAS; n = 6) [33, 34, 37, 42, 49, 52], the Health Survey Short Form 36 (SF-36; n = 4) [34, 37, 42, 53], and the Pain Self-Efficacy Questionnaire (PSEQ; n = 4) [51, 54, 56, 59]. These four tools represent reliable and widely-used measures of low back pain disability [70], intensity and frequency of chronic pain [71], health status [72] and ability to cope and manage despite pain [73, 74] respectively.

Changes in medication use before and after interventions were reported by seven studies [35, 37, 40, 43, 53, 56, 57], with only three of these delivering medication education [35, 37, 56]. One study by Sullivan and colleagues (2017) was specifically aimed at tapering opioid use [56].

### Risk of bias and quality of reporting

**Fig 2:**
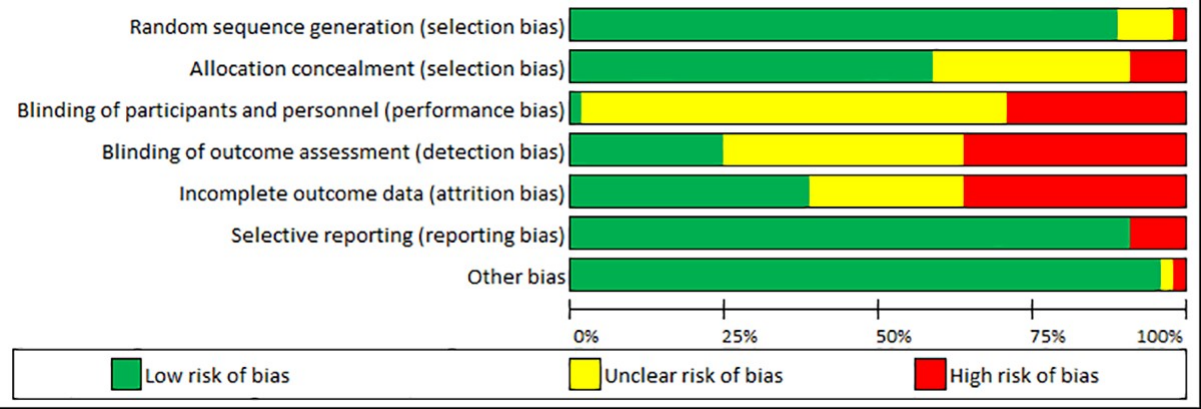
Risk of bias bar graph.

Risk of bias (Table 4 and Fig 2) was variable across all seven Cochrane Risk of Bias elements, with Kerns et al. 2014 [48], reporting the lowest risk of bias for all elements.

**Table 4:** Risk of bias assessment.

Authors	Random Sequence generation (selection bias)	Allocation concealment (selection bias)	Blinding of participants and personnel (performance bias)	Blinding of outcome assessment (performance bias)	Incomplete outcome data (attrition bias)	Selective reporting (reporting bias)	Other bias
Abbasi *et al*. 2012 [33]							
Anderson *et al*. 2007 [34]							
Bair *et al*. 2014 [35]							
Basler *et al*. 1996 [36]							
Becker *et al*. 2000 [37]							
Beltran-Alacreu *et al*. (2015) [38]							
Burke *et al*. (2016) [39]							
Chao *et al*. (2019) [59]							
Cherkin *et al*. (2001) [40]							
Corson *et al*. (2011) [41]							
Cramer *et al*. (2013) [42]							
Ersek *et al*. (2008) [43]							
Gallagher *et al*. (2013) [44]							
Heutink *et al*. (2012) [45]							
Jay *et al*. (2016) [46]							
Keller *et al*. (1997) [47]							
Kerns *et al*. (2014) [48]							
Kristjánsdóttir *et al*. (2013) [49]							
Martin *et al*. (2012) [50]							
Nicholas *et al*. (2013) [51]							
Pires *et al*. (2015) [52]							
Ribero *et al*. (2008) [53]							
Ryan *et al*. (2010) [54]							
Smith *et al*. (2016) [55]							
Sullivan *et al*. (2017) [56]							
Turner-Stokes *et al*. (2003) [57]							
Uebelacker *et al*. (2016) [58]							

Low risk of bias is shaded green; High risk of bias is shaded red; Unclear risk of bias is shaded yellow

All but three [43, 47, 59] of the included studies showed adequate randomisation of study participants: 13 studies reported random number generation [34, 36, 38–41, 44–46, 50, 54, 57, 58]; eight studies used block randomisation [33, 35, 37, 42, 48, 49, 52, 55]; and one study each reported drawing lots [53], a mix of block randomisation and random number tables [51] and a mix of computer-generated randomisation, sealed envelopes and blocked randomisation [56]. The three remaining studies involved biased randomisation in pain centres that could not be paired [43], did not specify how randomisation was achieved [47], or adopted a non-randomised design [59].

Blinding of participants and personnel (i.e. performance bias), blinding of outcome assessment (i.e. detection bias), and incomplete outcome data (i.e. attrition bias) showed the highest risk of bias across studies. For example, nine studies did not report complete outcome data as stated in their respective protocols and/or methodology [34, 36, 39, 44, 53–55, 57, 58]. Allocation concealment (i.e. selection bias) was deemed high risk in three included studies [57–59] and techniques used for concealment were unclear for nine studies [33, 34, 36, 41, 43–45, 47, 50].

Reporting bias and other bias were generally low across all 27 studies. Based on the information outlined in study protocols, three studies showed selective outcome reporting [33, 35, 55]. Other than a potential conflict of interest in one study [54] and design issues in one non-randomised study [59], no additional sources of bias were identified.

**Fig 3:**
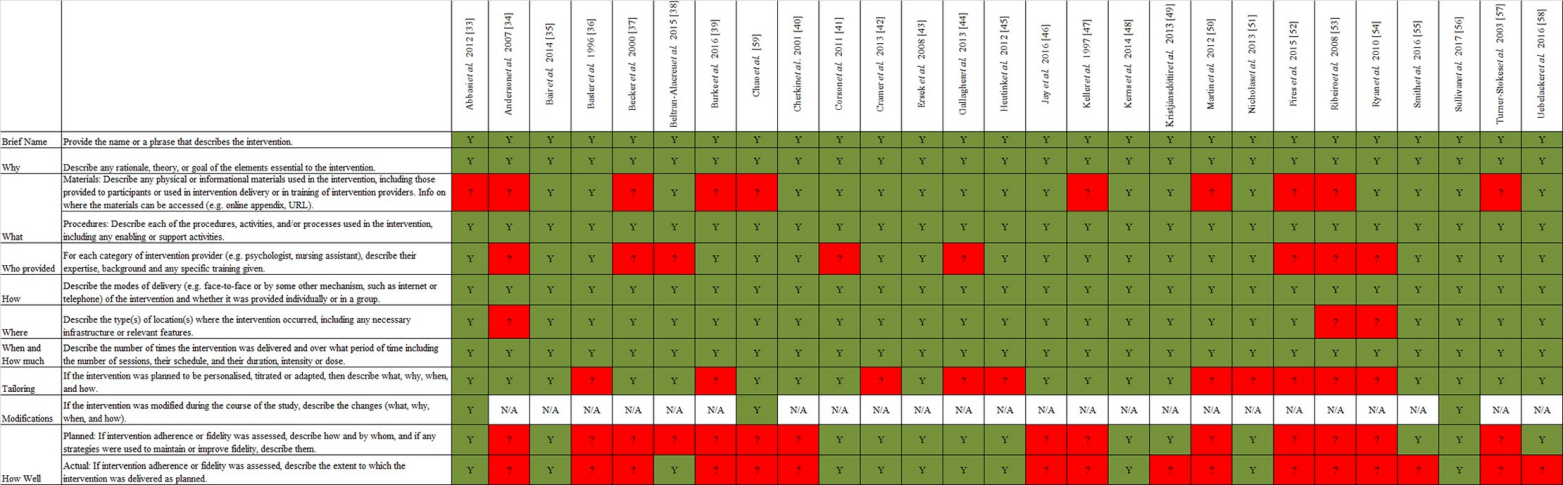
TIDieR table for quality of reporting.

Generally, the quality of reporting was satisfactory when assessed against the TIDieR checklist [66, 67] (Fig 3). On average, eight items were reported across all 27 included studies. One study [56] reported all 12 checklist items, while another [35] reported all 11 checklist items as it did not involve any modifications during the course of study. All included studies described five of the 12 checklist items (i.e. brief names; why; procedures; how; and when and how much) in their methods sections. Intervention location was the next best reported checklist item, with only three studies [34, 53, 54] not providing details of where they were conducted. Modifications were made and reported by three studies [33, 56, 59]. Planned and actual fidelity assessments were the least reported checklist items, with 14 studies not describing plans for determining fidelity [34, 36–40, 46, 47, 50, 52–54, 57, 59] and 16 studies not reporting if any fidelity assessment plans were followed through [34, 36, 37, 39, 40, 46, 47, 49, 50, 52–55, 57–59].

### Summary of findings

Overall, education as part of MD interventions, either as the active intervention or the comparator, seemed to show positive outcomes for participants. These positive outcomes generally appeared to be sustained or improved at follow-up. Topics around physical activity and cognitive-behavioural strategies were most common. The majority of included studies offered take-home materials in the form of written supplements and homework to help participants re-visit content at their own convenience. Interventions were conducted as one-on-one and/or group weekly didactic sessions, targeting any type of chronic pain. MD teams across all included studies consisted mostly of physiotherapists, psychologists and nurses. However, variable risks of bias and heterogeneity in outcome measures were observed across all 27 included studies.

## Discussion

This systematic review identified that education, as part of MD interventions, appeared to contribute to an immediate amelioration in chronic pain management, with benefits seemingly sustained or improved long-term. This was despite the observed heterogeneity in: participant type and duration of chronic pain; study methods; outcome measures; and quality of reporting. Commonalities identified across the included studies provide important insights to all relevant stakeholders about potentially successful strategies for future adoption.

### Common aspects across included studies and deductions

Most education sessions were run in groups, for a minimum of two hours each, over a four- to ten-week period. They incorporated direct, face-to-face interactions between educators and participants, and provided take-home materials and homework activities. These characteristics all seemed to optimise group outcomes and brought positive changes to participants’ lifestyles and quality of life over time, a finding similar to that reported in a review of cancer pain management [75]. Duration of education sessions also aligned with previous recommendations of effective instruction time of 2.5 hours for low back pain [76].

In terms of skill sets of recruited healthcare professionals, physiotherapists, psychologists and nurses were the most common intervention facilitators, potentially indicative of a need to focus on education around physical activity, mental health (notably, cognitive behavioural strategies) and general lifestyle changes. Communication between participants and these three types of healthcare professionals is likely to reshape beliefs and behaviour of people with chronic pain, an outcome identified in previous research involving various healthcare professionals and health conditions such as low back pain and palliative care [28, 77–80].

### Directions for future research

Moving forward, researchers should incorporate all fore-mentioned education characteristics in future RCTs of MD programs. Additionally, future RCTs should: expand on the MD team; use only validated outcome measures; extend follow-up periods to investigate the longitudinal effects on participants and health systems; and analyse the effectiveness of chronic pain management strategies in developing and non-developing countries.

#### 1. Expanding the MD team

Almost half of the included studies (n = 11) did not report on the full composition of their MD teams, thereby creating a lack of transparency around the exact range and diversity of professionals involved. Chronic pain, by its very nature, is a complex medical condition that could require a broad array of healthcare professionals for optimal management. Research on the impact of bigger and more diverse MD teams than those used historically are recommended. The immediate and long-term impact of such teams on participants (e.g. the inclusion of GPs, pain specialists, pharmacists, psychologists, physiotherapists, nurses, nutritionists, occupational therapists and social workers), can only be hypothesised at this stage, as highlighted by previous preliminary studies [25, 26].

Involvement of pharmacists in only two interventions was surprising and might indicate that these professionals are overlooked in chronic pain management, despite them being strategically placed in primary care to monitor, advise, support and refer consumers with chronic pain [9]. Future RCTs exploring the role of pharmacists in MD chronic pain management, especially in terms of medication management and counselling, are warranted to further investigate the positive contributions of these professionals [25, 26].

A number of other healthcare professionals were overlooked in MD chronic pain management; occupational therapists, nutritionists and social workers were involved in only one study each. These healthcare professionals have an important role in providing assistance to patients with chronic pain. Occupational therapists can assist patients to reach optimal health and well-being through participation in everyday activities [81]. Nutritionists can educate patients on the impact of food in reducing chronic inflammation, a symptom associated with chronic pain, as well as assist with optimal weight management [82]. Social workers can establish, monitor and improve practice and ethical standards within MD teams, as well as advise patients on relevant and available services, such as subsidised healthcare plans [83]. Future RCTs investigating MD teams that also involve these healthcare professionals, are warranted.

#### 2. Using only validated outcome measures

Instead of combined use of validated and non-validated outcome measures (as observed across most studies in this review), it is recommended that only evidence-based measures, such as the Pain Self-Efficacy Questionnaire (PSEQ), Assessment of Quality of Life-6D (AQoL-6D) and Patient Global Impression of Change (PGIC), be utilised in future RCTs. These represent reliable and consistent ways of assessing participants’ ability to physically and emotionally cope with and manage pain over time [74, 84–90]. Given that VAS, RMDQ and SF-36 detected significant changes [33–35, 37, 42, 43, 49, 51–54] in this review, these tools are recommended for similar interventions in the future. Consistent use of only validated outcome measures will facilitate comparison of outcomes across studies. Only then, will other systematic frameworks, such as Grading of Recommendations Assessment, Development and Evaluation (GRADE) [91], be used effectively to make clinical practice recommendations.

#### 3. Extending follow-up periods to assess longitudinal effects

This review has only reported positive, short-term (less than two years) lifestyle benefits of education, as part of MD programs, in individuals experiencing multiple types of chronic pain. Yet, longitudinal lifestyle, behavioural changes and economic impact (e.g. in the form of savings incurred to participants, health systems and the economy) over multiple years, remain other aspects to be investigated. The authors propose an extension of follow-up periods to analyse these effects.

#### 4. Conducting RCTs in developing and non-developing countries

Despite a high proportion of the population in developing countries reporting chronic pain [4, 92], this review has mostly considered studies from developed nations (n = 25). Local traditions, cultural background and/or local policies tend to vary among developed, developing and non-developing countries, and can significantly influence diagnosis and treatment of chronic pain in these areas [10, 92]. With an inevitably ageing population and a predictable rise in prevalence of chronic pain globally [5, 29], investigating the provision of cost-effective MD programs that incorporate education in developing and non-developing countries is another avenue for future research. Only then, can chronic pain be truly and effectively managed on a global scale.

### Strengths and limitations

The initial aim of this review was to update the work of Scascighini *et al*. [27]. Despite multiple attempts to contact the authors, a detailed search strategy of the 2008 review was not obtained. Consequently, an alternative up-to-date search strategy, aimed at better identifying relevant articles, was developed. While the total number of studies included in this work (n = 27) was not dissimilar to the 35 and 31 studies analysed by Scascighini *et al*. and Berube *et al*. respectively [27, 30], this work set out with a broader focus of exploring the effects of any type of education, delivered as part of MD interventions, on any chronic pain type. Inclusion of all possible forms of education was a strength of this review and addressed the authors’ original objective of reporting on the ideal combination of education methods to optimise self-management of chronic pain.

Another strength was the use of several databases to identify relevant studies, a strategy previously identified as broadening the scope of research and improving the quality of systematic reviews [93]. Setting language limits to English, French, Dutch and German, rather than English alone, added to this strength by capturing a wider range of articles and increasing the generalisability of conclusions. Comprehensive assessments of methodological quality and risk of bias, as well as analysis of longitudinal effectiveness in relation to follow-up periods [94], further consolidated conclusions of this review. These strategies were seldom reported in earlier work [27, 30–32].

Conversely, heterogeneity of included studies was a limitation as it made application of the Cochrane Collaboration Risk of Bias tool [63–65] and the TIDieR model [66, 67] difficult. The comparative process proved especially challenging due to the diversity in individual studies in terms of: 1. inclusion criteria for participants, especially in terms of duration of their pain; 2. education strategies adopted; 3. the array of validated and non-validated or customised assessment tools utilised to monitor participant progress; and 4. the variety of healthcare professionals involved. Additional inherent limitations of the TIDieR tool also imply potential uncertainty in the determination of quality of intervention reporting and reproducibility of specified methods [95]. As stipulated by Cotterill *et al*. (2018), TIDieR limits its quality and reproducibility assessments only to the context or setting specified by the individual study analysed, thereby failing to provide an overview of quality and reproducibility of similar studies in different contexts or settings over time [95].

Another limitation was restriction of the authors’ search strategy to only four databases and four languages, which implied that other relevant studies may have been missed. No grey literature was searched and it is likely that studies identifying negative effects of education may not have been reported.

### Gaps in existing literature

Interpretation of results from this review calls for caution as variable risks of bias, uncertainties around quality, and lack of consistency (e.g. in terms of MD teams and outcome measures), were reported as shortcomings of included studies. There is an increased need for greater homogeneity in research methodology, such as use of intention-to-treat, adequate concealment and consistent use of only validated outcome measures. Detailed reporting of interventions to ease reproducibility, for example in terms of: type of professional recruited; type of materials used; and intervention adherence measures, is also required. Clarity around participant characteristics such as exact duration of chronic pain, will further increase reliability and generalisability of any findings promoting self-management and self-efficacy in people with this condition. Lastly, given the high proportion of studies from developed nations, conclusions from this review may not be generalised to people with chronic pain in developing and non-developing nations.

## Conclusion

Participant education remains an understudied aspect of MD interventions focused on chronic pain management. Compared to previous work, this review has attempted to present reliable and comprehensive evidence around the effects of including education in such programs. MD programs that include four- to ten-week education around topics of physical activity, cognitive behavioural strategies and general lifestyle, generally point towards significantly positive results, particularly in relation to self-management practices and self-efficacy. Such programs should be adopted as part of a patient’s pain management plan in the future. However, some research gaps remain, including a lack of consistency in reporting participant chronic pain duration; use of standardised and validated outcome measures in combination with non-validated measures; and lack of homogeneity in research methodology. A need for more research that expands on the MD team, uses only recommended outcome measures, extends follow-up periods and is implemented in various developing and non-developing countries, is warranted.

## Supporting information

S1 Appendix Preliminary database search strategy. (DOCX)

S2 Appendix Database search strategy. (DOCX)
