# Supplementary material for: Multi-disciplinary interventions for chronic pain involving education: A systematic review
Source: PLoS One. 2019 Oct 2;14(10):e0223306. doi: 10.1371/journal.pone.0223306 (PMC6774525; doi:10.1371/journal.pone.0223306)
Supplement: S1 Appendix — (DOCX) [file pone.0223306.s001.docx]

**S1 Appendix. Preliminary database search strategy.**

The following databases were searched to identify and analyse previously-conducted systematic reviews:

1. PubMed
2. Cochrane
3. GoogleScholar

Date of search: July-August 2017

1. **PubMed Search Parameters**

[[Chronic Pain AND Review] in title] AND [Education] in abstract

Limit: 2007-2017

10 hits

1. **Cochrane Search Parameters**

#1 pain clinic

#2 interdisciplinary communication

#3 patient education as topic

#4 chronic pain

All terms were identified as MeSH and exploded to keep the search broad

#1 AND #2 AND #4 revealed one hit; #1 AND #2 AND #3 AND #4 revealed no hit

1. **GoogleScholar Search Parameters**

“Multidisciplinary intervention chronic pain systematic review”

1 hit identified as relevant i.e. Scascighini *et al.*, 2008.
