# Supplementary material for: Multi-disciplinary interventions for chronic pain involving education: A systematic review
Source: PLoS One. 2019 Oct 2;14(10):e0223306. doi: 10.1371/journal.pone.0223306 (PMC6774525; doi:10.1371/journal.pone.0223306)
Supplement: S2 Appendix — (DOCX) [file pone.0223306.s002.docx]

**S2 Appendix. Database search strategy.**

The following databases were searched to analyse multi-disciplinary interventions, in the form of RCTs and/other controlled studies, involving education:

1. CINAHL
2. Embase
3. PsycInfo
4. PubMed

Date of search: Mid-2017 to August 2019

1. **CINAHL Search**

The search strategy was to combine the following MeSH terms:

- S1: “Chronic Pain”
- S2: “Health Education”
- S3: “Pain Management”
- S4: “Pain Clinics”
- S5: “Self Care”
- S6: “Multidisciplinary Care Team”
- S7: “Randomised Controlled Trial” OR “Clinical Trials”

Combination of MeSH terms was as follows:

- S8: “S1 AND S2”
- S9: “S1 AND S3”
- S10: “S1 AND S4”
- S11: “S1 AND S5”
- S12: “S1 AND S6”
- S13: “S1 AND S7”
- S14: “S8 AND S13”
- S15: “S9 AND S13”
- S16: “S10 AND S13”
- S17: “S11 AND S13”
- S18: “S12 AND S13”

No limits set

No. of hits = 42

1. **Embase Search**

The search strategy was as follows:

- #1: ‘chronic pain’/exp
- #2: ‘health promotion’/exp
- #3: ‘patient education’/exp
- #4: ‘interdisciplinary communication’/exp
- #5: ‘interdisciplinary education’/exp
- #6: ‘self care’/exp
- #7: ‘intervention study’/exp
- #8: ‘controlled clinical trial’/exp
- #9: #2 OR #3
- #10: ‘pain clinic’/exp
- #11: #4 OR #5 or #10
- #12: #7 OR #8
- #13: #1 AND #9
- #14: #1 AND #11
- #15: #1 AND #6
- #16: #1 AND #12
- #17: #13 AND #16
- #18: #14 AND #16
- #19: #15 AND #16

No limits set

No. of hits = 229

1. **PsycInfo Search**

The search strategy was as follows:

- 1 exp Chronic Pain/
- 2 exp Health Education/
- 3 exp Interdisciplinary Treatment Approach/
- 4 exp Pain Management/
- 5 exp Self-Management/
- 6 4 or 5
- 7 1 and 2
- 8 1 and 3
- 9 1 and 6
- 10 exp Clinical Trials/
- 11 exp Treatment Effectiveness Evaluation/
- 12 10 or 11
- 13 7 and 12
- 14 8 and 12
- 15 9 and 12

No limits set

No. of hits = 151

1. **PubMed Search**

The search strategy was as follows:

- #1 chronic pain[MeSH Terms]
- #2 health education[MeSH Terms]
- #3 pain management[MeSH Terms]
- #4 self-management[MeSH Terms]
- #5 pain clinics[MeSH Terms]
- #6 patient care team[MeSH Terms]
- #7 controlled clinical trial[MeSH Terms]
- #8 (chronic pain[MeSH Terms])AND health education[MeSH Terms]
- #9 (pain management[MeSH Terms]) OR self-management[MeSH Terms]
- #10 (chronic pain[MeSH Terms]) AND ((pain management[MeSH Terms])

OR self-management[MeSH Terms])

- #11 (pain clinics[MeSH Terms]) OR patient care team[MeSH Terms]
- #12 (chronic pain[MeSH Terms]) AND ((pain clinics[MeSH Terms]) OR

patient care team[MeSH Terms])

- #13 (((chronic pain[MeSH Terms])AND health education[MeSH Terms]))

AND controlled clinical trial[MeSH Terms]

- #14 (((chronic pain[MeSH Terms]) AND ((pain management[MeSH Terms])

OR self-management[MeSH Terms]))) AND controlled clinical

trial[MeSH Terms]

- #15 (((chronic pain[MeSH Terms]) AND ((pain clinics[MeSH Terms]) OR

patient care team[MeSH Terms]))) AND controlled clinical trial[MeSH

Terms]

No limits set

No. of hits = 63
